# Streptococcal superantigen‐induced expansion of human tonsil T cells leads to altered T follicular helper cell phenotype, B cell death and reduced immunoglobulin release†

**DOI:** 10.1111/cei.13282

**Published:** 2019-03-13

**Authors:** F. J. Davies, C. Olme, N. N. Lynskey, C. E. Turner, S. Sriskandan

**Affiliations:** ^1^ Department of Medicine Imperial College London London UK; ^2^ Present address: Department of Molecular Biology and Biotechnology University of Sheffield Sheffield UK

**Keywords:** antibody, group A streptococcus, SpeA, streptococcal pyrogenic exotoxin A, streptococcus pyogenes

## Abstract

Streptococcal pyrogenic exotoxin (Spe) A expression is epidemiologically linked to streptococcal tonsillo‐pharyngitis and outbreaks of scarlet fever, although the mechanisms by which superantigens confer advantage to *Streptococcus pyogenes* are unclear. *S. pyogenes* is an exclusively human pathogen. As the leucocyte profile of tonsil is unique, the impact of SpeA production on human tonsil cell function was investigated. Human tonsil cells from routine tonsillectomy were co‐incubated with purified streptococcal superantigens or culture supernatants from isogenic streptococcal isolates, differing only in superantigen production. Tonsil cell proliferation was quantified by tritiated thymidine incorporation, and cell surface characteristics assessed by flow cytometry. Soluble mediators including immunoglobulin were measured using enzyme‐linked immunosorbent assay. Tonsil T cells proliferated in response to SpeA and demonstrated typical release of proinflammatory cytokines. When cultured in the absence of superantigen, tonsil preparations released large quantities of immunoglobulin over 7 days. In contrast, marked B cell apoptosis and abrogation of total immunoglobulin (Ig)A, IgM, and IgG production occurred in the presence of SpeA and other superantigens. In SpeA‐stimulated cultures, T follicular helper (Tfh) cells showed a reduction in C‐X‐C chemokine receptor (CXCR)5 (CD185) expression, but up‐regulation of OX40 (CD134) and inducible T cell co‐stimulator (ICOS) (CD278) expression. The phenotypical change in the Tfh population was associated with impaired chemotactic response to CXCL13. SpeA and other superantigens cause dysregulated tonsil immune function, driving T cells from Tfh to a proliferating phenotype, with resultant loss of B cells and immunoglobulin production, providing superantigen‐producing bacteria with a probable survival advantage.

## Introduction

The human pathogen *Streptococcus pyogenes* can produce up to 11 different secreted superantigens that contribute to the features of cytokine‐induced toxic shock during lethal, invasive infections such as necrotizing fasciitis 1. Invasive infections are, however, rare compared with symptomatic non‐invasive disease that occurs in the nasopharynx, manifest as pharyngitis, tonsillitis and the childhood exanthem scarlet fever. Indeed, in human populations, the throat and tonsils represent the main reservoir of *S. pyogenes* carriage.

When secreted in the vicinity of host leucocytes, streptococcal superantigens bind host major histocompatibility complex II (MHC‐II) outside the antigen groove and ligate a variably discrete repertoire of T cell receptor variable β chain (TCR‐Vβ) subunits, thereby leading to mass activation and proliferation of all target populations of T cells that bear relevant TCR‐Vβ 2. As such, the evolutionary benefit of superantigen production is most probably conferred to *S. pyogenes* through activation of T cells within the nasopharynx and, in particular, the human tonsil, in ways that provide a survival or transmission advantage.

The tonsil is a solid secondary lymphoid organ that possesses only efferent lymphatic drainage; the leucocyte populations that constitute the tonsil are distinct from those present in peripheral blood and also distinct from mucosal lymphoid tissue. The tonsil comprises follicular dendritic cells, T follicular helper (Tfh) cells and B cells arranged in germinal centres, bounded by the specialized tonsil mucosal epithelium in the posterior nasopharynx 3.

Streptococcal expression of superantigen genes is increased upon exposure to tonsil epithelium 4 and models of tonsillo‐pharyngitis 5. Furthermore, the phage‐encoded superantigen streptococcal pyrogenic exotoxin A (SpeA) is required for successful nasopharyngeal infection with *S. pyogenes* in human leucocyte antigen (HLA)‐DQ8 superantigen‐sensitive transgenic mice, albeit that mice lack tonsils 6. It is therefore remarkable that, in contrast to work that has elucidated effects of superantigens on rodent spleen cells and leucocytes of human peripheral blood 1, the impact of superantigens on human tonsil has not been addressed. Using the phage‐encoded toxin SpeA, which has been implicated in the emergence of severe *S. pyogenes* infections in recent decades, as a paradigm of a streptococcal superantigen 7 we set out to conduct a systematic examination of responses to superantigen using both human tonsil histocultures and cultured cell suspensions.

## Materials and methods

### Reagents

Recombinant toxins were purchased from Toxin Technology (Sarasota, FL, USA). Recombinant streptococcal mitogenic exotoxin Z (SmeZ) and Streptococcal pyrogenic exotoxin J (SpeJ) were a kind gift of Thomas Proft (Auckland, NZ), 8. Concanavalin A (Sigma, Welwyn Garden City, UK), anti‐CD3 and anti‐CD28 (Miltenyi Biotech, Woking, UK) were used at 1 μg/ml, as per the supplier’s recommendations. Anti‐cytokine antibodies were purchased from R&D Systems (Abingdon, UK). Antibodies for flow cytometry were purchased from BD Biosciences (Swindon, UK), conjugated to either fluorescein isothiocyanate (FITC), phycoerythrin (PE), PE cyanin 5 (PECy5) or peridinin chlorophyll (PerCP)Cy7, with the following exceptions: TCR‐Vβ antibodies, isotype controls and TCR‐Vβ repertoire kit (IOTest Beta Mark) from Beckman Coulter (Marseilles, France), CXCR5 PE and isotype control from R&D Systems, CXCR5 PE‐Cy5 and isotype control from eBioscience (High Wycombe, UK).

### Bacterial strains

Isogenic *emm*1/M1 *S. pyogenes* isolates that differ in SpeA production 9 or isogenic *emm*89/M89 isolates that differ in SmeZ production 10 were cultured overnight in RPMI supplemented with 10% fetal bovine serum (FBS) and cell‐free supernatants prepared as previously reported 11.

### Human tonsil donors and ethics

Fresh tonsils were obtained by the Imperial College Healthcare NHS Trust Tissue Bank (ICHTB) from anonymized adult donors (aged > 16 years) undergoing routine tonsillectomy and consenting to the use of tissue surplus to diagnostic requirement for research. The use of donor tissue was approved by a Research Ethics Committee (07/Q0407/38) and by the Tissue Management Committee of the ICHTB (R12011). Donors (53 females; 12 males) had a median age of 28 years (range = 17–59). Indication for tonsillectomy was prolonged or recurrent tonsillitis in all cases, with the exception of four males with sleep apnoea. Other medical and demographic data were not available.

### Tonsil cell suspensions and histocultures

Tonsil cell suspensions and histocultures were prepared according to previously published methods 12 with modification to include kanamycin 100 µg/ml and amphotericin 2·5 µg/ml. Cells were co‐incubated with different concentrations of purified superantigen or filter‐sterilized bacterial supernatant. At the end of experiments, tonsil cells were harvested into sterile tubes, centrifuged at 300 ***g*** for 10 min, and tonsil supernatant collected and stored at –20°C. Tonsil cells were either used immediately or stored at –80°C in Freezimix (90% fetal calf serum; Invitrogen, Basingstoke, UK) and 10% dimethylsulphoxide (DMSO); Sigma) for later flow cytometry analysis. For histocultures, the capsule, connective tissue and any damaged tissue was removed from tonsil and remaining tissue dissected into 2–3‐mm blocks then placed on 1 × 1 × 2 cm Gelfoam constructs (Upjohn, Pfizer, Maidenhead, UK). Histoculture supernatants were collected from the Gelfoam constructs at the end of the experiment and stored at –20°C.

### Cellular and immunological assays

Soluble mediators in cell‐free tonsil supernatants were measured by enzyme‐linked immunosorbent assay (ELISA) [immunoglobulin enzyme immunoassay (EIA); Bethyl Labs, Montgomery, TX, USA; cytokine Duosets EIA kits, R&D Systems, Abingdon, UK]. Tonsil cell proliferation was measured by [^3^H]‐labelled tritium incorporation and TCR‐Vβ subsets were evaluated by flow cytometry. Human tonsil cells were separated using AutoMACS Bead Technology (Miltenyi Biotech), either before or after culture. T cells were isolated using CD2‐positive selection. B cells were then isolated using the B cell‐negative selection kit, which positively selected for CD2, CD14, CD16, CD36, CD43 and CD235a, leaving only B cells remaining. Confirmation of the quality of separation was performed using surface staining for CD3 (T cells) and CD20 (B cells), and gave > 99% cell purity on each occasion. Tonsil cell migration in response to CXCL13 was evaluated using the Transwell technique, as previously described 13, using tonsil cells preincubated with SpeA 1000 ng/ml or medium only for 6 days, and 100 nM of CXCL13, a concentration approximately five times that secreted by unstimulated tonsil cell cultures. After 1 h, cell suspensions in the lower chamber were aspirated and stained using CD4‐FITC or CD4‐PE/Cy5 and CXCR5‐PE. Migrated CD4^+^CXCR5^+^ cells were enumerated using Countbright beads (Invitrogen) and known percentage positivity in each sample in triplicate. Chemotactic index was calculated by dividing the mean number of cells migrating towards CXCL13 by the number of cells migrating towards a buffer control.

### Statistics

All statistical analyses, unless stated specifically elsewhere, were performed using Graphpad Prism version 5 software. All data were non‐parametric; two group comparison Mann–Whitney tests were performed, or Wilcoxon’s signed‐ranked test for paired data and two‐way analysis of variance (anova) for multiple grouped data (migration assay and bacterial supernatant IgG). For comparison of multiple groups, a one‐way anova (Kruskal–Wallis, or Friedman for paired data) test was performed. Statistical tests were not performed on groups with a sample size smaller than three. A probability value < 5% was classed as significant (*P* < 0·05).

## Results

### Tonsil T cells proliferate in response to superantigens and release proinflammatory cytokines

**Figure 1:**
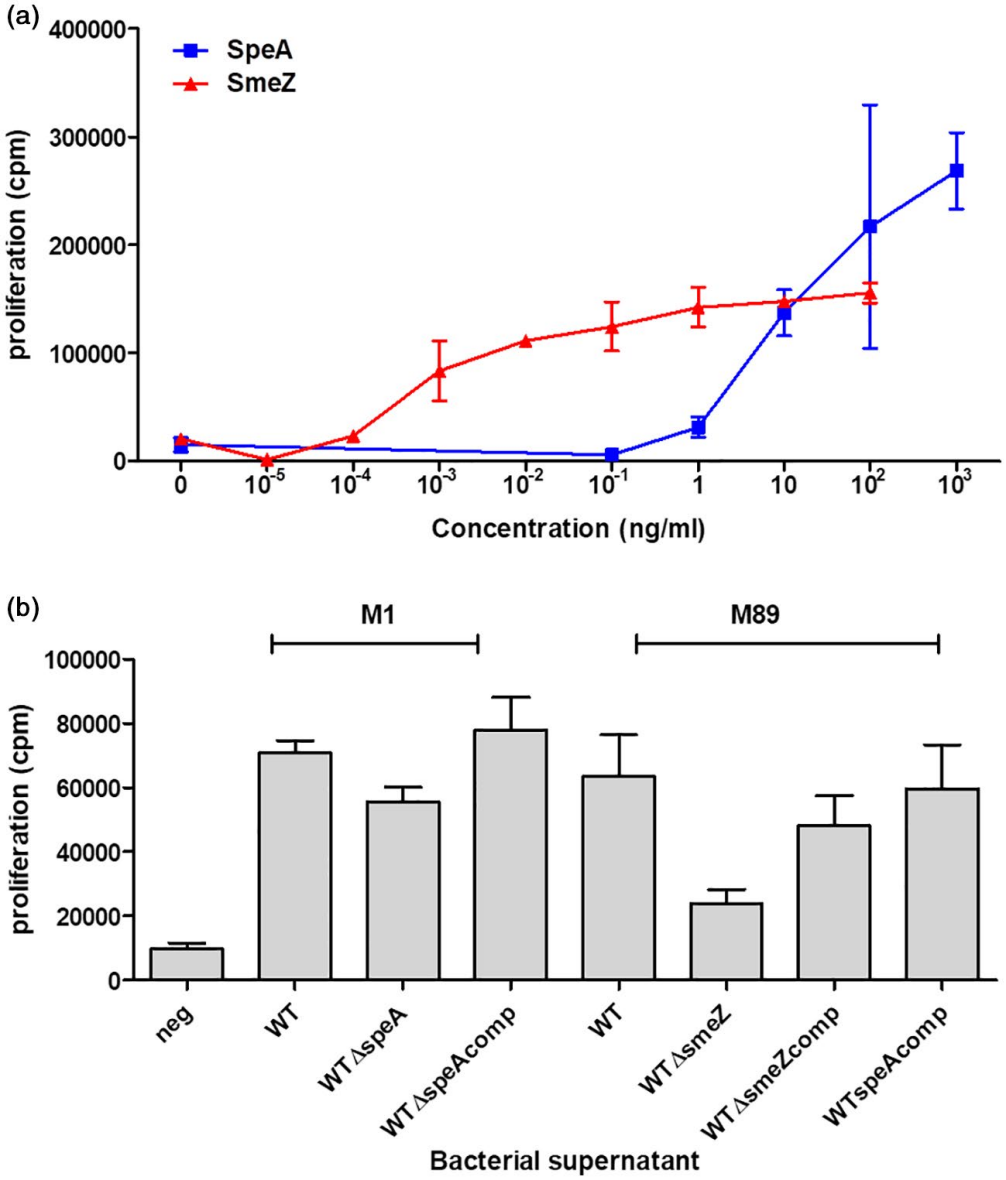
Tonsil cell proliferation in response to purified streptococcal superantigens or streptococcal supernatants. Tonsil cells were stimulated for 7 days with different concentrations of purified superantigens streptococcal pyrogenic exotoxin (SpeA) or streptococcal mitogenic exotoxin Z (SmeZ) (a) or 1% supernatants from isogenic *emm*1 or *emm*89 strains (b). *emm*1 supernatants were from parent [wild‐type (WT)]; isogenic *speA* negative (WTΔspeA); and complemented (WTΔspeAcomp) strains. *emm*89 supernatants were from parent (WT); isogenic *smeZ*‐negative (WTΔsmeZ); complemented (WTΔsmeZcomp); and SpeA‐complemented (WTspeAcomp) strains. Unstimulated control cells (neg) were incubated with medium alone. Data show one donor with mean and experimental triplicates; representative of six different tonsil donors for supernatants (*P* = 0·0047, Friedman test).

To establish that fresh human tonsil cell suspensions were responsive to streptococcal superantigens, cells were exposed to purified superantigens SpeA and SmeZ), and proliferation assessed by tritiated thymidine incorporation. Human tonsil cells demonstrated dose‐responsive proliferation similar to that seen with peripheral blood mononuclear cells (Fig. 1a). Specific superantigen responses were also seen using cell‐free bacterial culture supernatants from isogenic *emm*1 and *emm*89 streptococcal strains differing only in SpeA or SmeZ expression, respectively (Fig. 1b). Characteristic expansion of TCR‐Vβ T cell subsets was demonstrated in tonsil cells, most clearly in response to SmeZ (Supporting information, Fig. 1), due to the high proportion of TCR‐Vβ8 cells present in unstimulated tonsil preparations.

**Figure 2:**
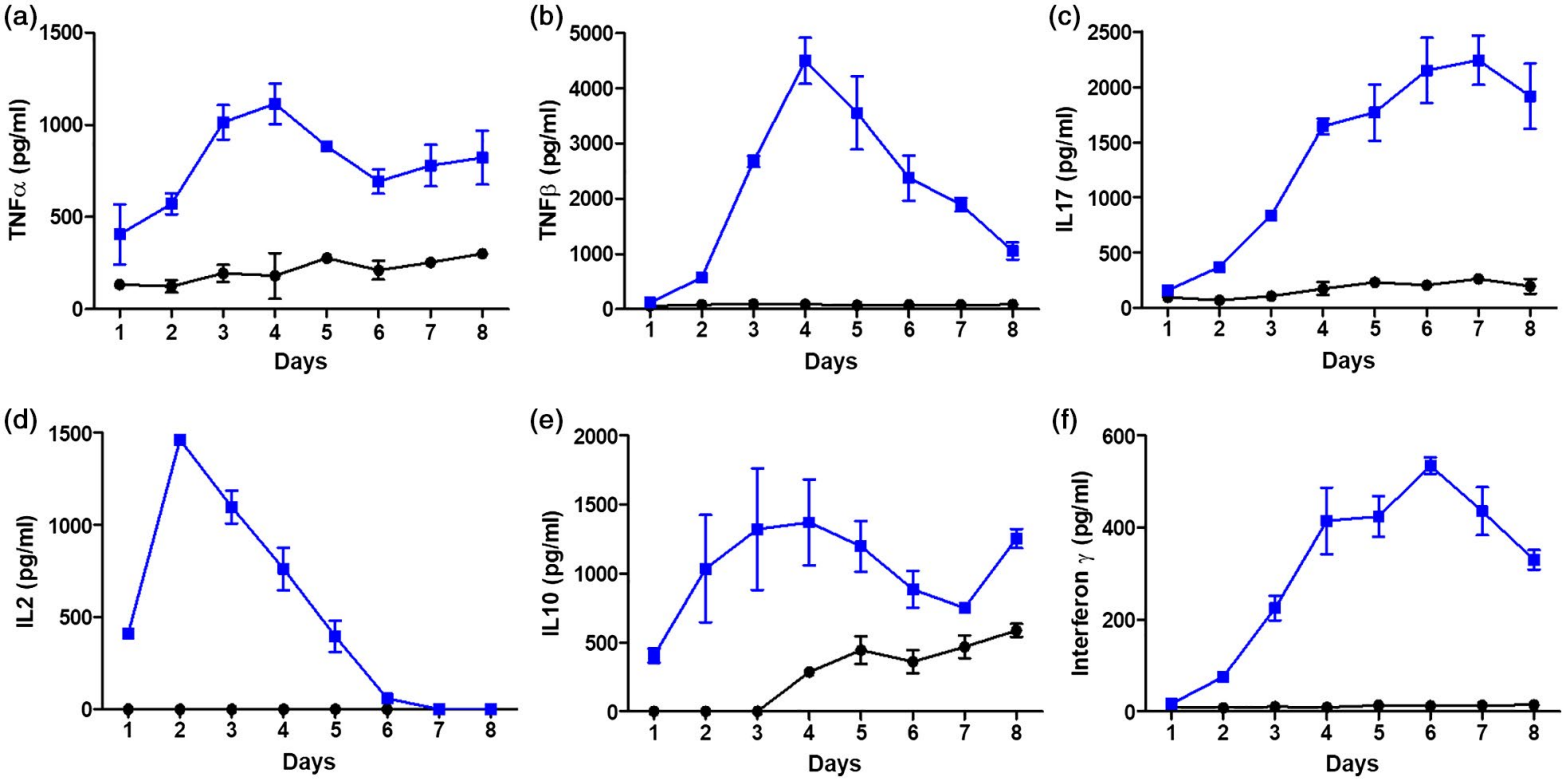
Streptococcal pyrogenic exotoxin (SpeA) induces proinflammatory cytokine release by stimulated tonsil T cells. Tonsil cells were stimulated for 1 week with recombinant SpeA 100 ng/ml (blue) or unstimulated control (black) and supernatants harvested daily from days 1 to 8 of culture. Harvested supernatants were then analysed by enzyme‐linked immunosorbent assay (ELISA) for the presence of cytokines tumour necrosis factor (TNF)‐α (a), TNF‐β (b), interleukin (IL)‐17 (c), IL‐2 (d), IL‐10 (e) and interferon (IFN)‐γ (f). Mean and standard deviation of experimental triplicates for a single representative donor is shown for each cytokine, representative of between five and 12 different donors measured for each cytokine (Table 1).

Tonsil cells stimulated with SpeA produced a range of proinflammatory cytokines over 7 days compared to unstimulated controls, where there was minimal cytokine production (Fig. 2 and Table 1). Production of interleukin (IL)‐2 by stimulated tonsil cells was rapid and preceded production of tumour necrosis factor (TNF)‐β (lymphotoxin‐α), which was followed by production of IL‐17 and interferon (IFN)‐γ. Taken together, the findings confirmed that explanted tonsil cell suspensions responded to streptococcal superantigens in a manner similar to previously demonstrated responses in peripheral blood.

**Table 1:** Peak cytokine production in unstimulated and SpeA‐stimulated tonsil cells

Cytokine	Peak day of production	Median unstimulated (pg/ml)	Median SpeA‐stimulated (pg/ml)	Number of tonsil donors	Significance§
IL‐2	2	30	938	5	*P* = 0·012
IL‐10	4	86	581	5	*P* = 0·036
IL‐17	4‐7	567	2241	7	*P* = 0·0156
TNF‐α	4	145	1054	12	*P* = 0·0005
TNF‐β	4	143	3616	8	*P* = 0·0078
IFN‐γ	5	10	535	7	*P* = 0·0156
TGF‐β	7	129	480	7	*P* = 0·0156

^§^ Interleukin (IL)‐2 and IL‐10 were assessed by Mann–Whitney *U*‐test, as numbers were too small for effective pairing; others were assessed using the Wilcoxon matched‐pairs signed‐rank test. SpeA = streptococcal pyrogenic exotoxin; TNF = tumour necrosis factor; IFN = interferon; TGF = transforming growth factor.

### Superantigen stimulation causes a loss of tonsil B cells and reduction in immunoglobulin production

**Figure 3:**
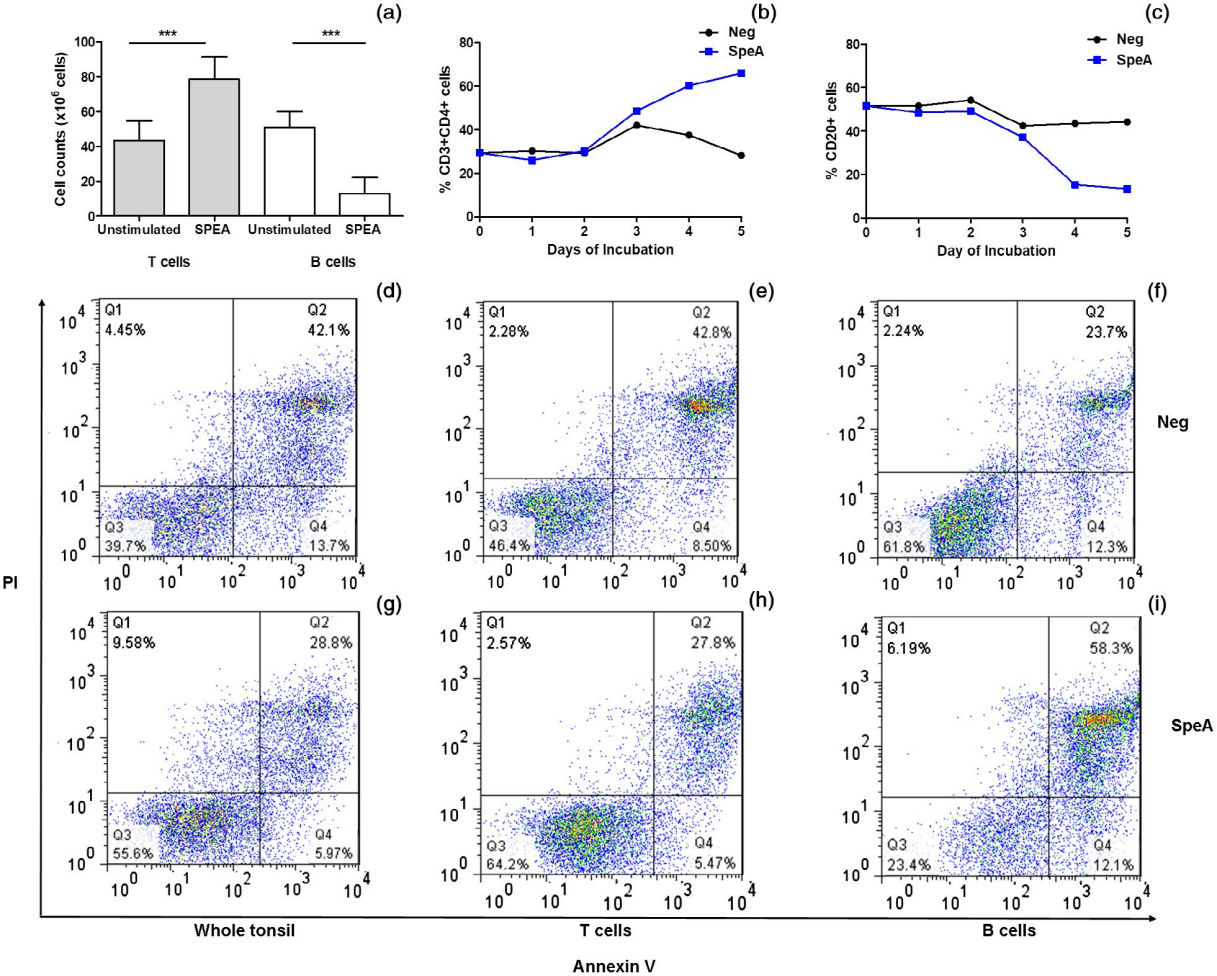
Streptococcal pyrogenic exotoxin (SpeA) results in expansion of tonsil T cells but apoptosis of B cells. Tonsil cells were analysed over 5 days’ incubation with SpeA 100 ng/ml. Compared to unstimulated controls, SpeA resulted in increased total T cell count but a decrease in the total B cell count (a) (*n* = 8 tonsil donors, *P* = 0·0039*** for T cells, *P* = 0·0078*** for B cells, both Wilcoxon’s paired signed‐rank *t*‐test). The proportions of CD3^+^CD4^+^ tonsil cells (b) increased in the presence of SpeA by day 3, while the reduction in B cells expressing CD20 (c) became apparent by day 4 (single donor time–course shown, representative of five donors). Annexin V and propidium iodide (PI) staining of the whole cell population showed an overall reduction in apoptosis in SpeA‐stimulated cells (28·8%) compared to unstimulated cells (42·1%) (d,g). After cell separation (autoMACS), T cells showed reduced apoptosis in the presence of SpeA (27·8 *versus* 42·8%) (e,h), while B cells showed a marked increase in apoptosis (58·3 *versus* 23·7%) (f,i), representative plot; two different tonsil donors.

Unstimulated tonsil cell suspensions comprise a mix of predominantly B and T cells, representative of the germinal centres present within tonsils. Despite expansion of the T cell population in tonsil cell suspensions over a 5‐day period (Fig. 3a,b), stimulation with SpeA caused a profound loss of B cells during the same time‐period, compared with unstimulated cells (Fig. 3a and 3c). B cells demonstrated apoptosis, as shown by increased expression of annexin V and propidium iodide (PI), with the change evident after 4–5 days of co‐culture with SpeA, in contrast to the expanded T cell population (Fig. 3d–i).

**Figure 4:**
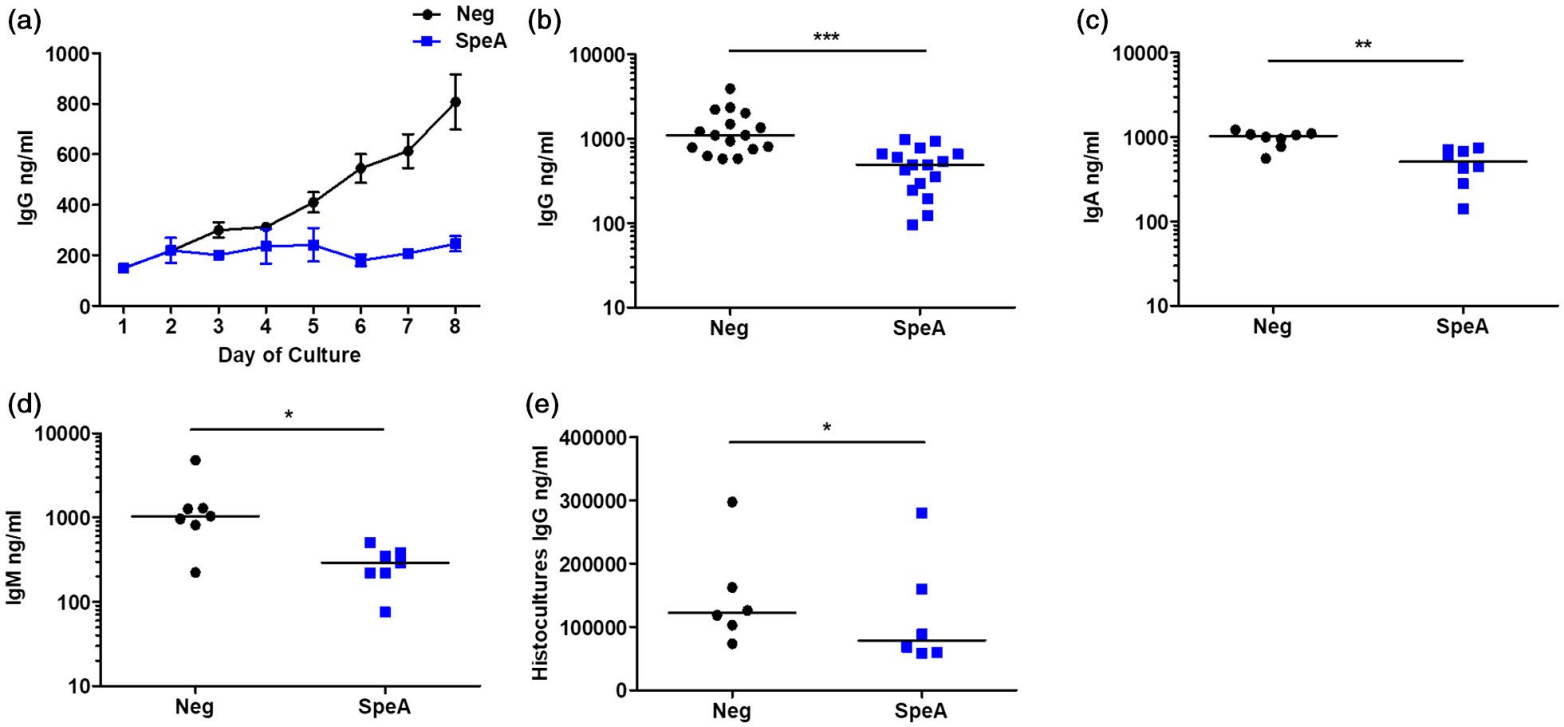
Reduction in tonsil cell immunoglobulin release in response to streptococcal pyrogenic exotoxin (SpeA). Tonsil cells were stimulated for 1 week with recombinant SpeA 100 ng/ml (blue) or unstimulated control (black) and supernatants harvested daily from days 1 to 8 of culture (a). Harvested supernatants were then analysed by enzyme‐linked immunosorbent assay (ELISA) for the presence of total immunoglobulin (Ig)G. Means and standard deviation (s.d.) of experimental triplicates from a single donor are shown at each time‐point, representative of five different donors. SpeA significantly reduced tonsil cell production of immunoglobulin (Ig)G (b, *n* = 16 donors, *P* = 0·0005***); IgA (c, *n* = 8 donors, *P* = 0·0078**); and IgM (d, *n* = 7 donors, *P* = 0·0156*) in comparison to unstimulated cultures, when a wider pool of donors was tested after 7 days of co‐incubation with SpeA. Horizontal bars represent median values. Non‐SpeA‐exposed control tonsil histocultures from seven donors produced large quantities of IgG (ng/ml), and this was diminished by co‐culture with SpeA (e) *P* = 0·014* (all Wilcoxon’s paired signed‐rank *t*‐test).

B cells in unstimulated tonsil cell culture began producing increased quantities of immunoglobulin (IgG, A and M) from day 4 of cell culture (Fig. 4). However, in SpeA‐stimulated cultures this response was abrogated, with no increase from baseline (Fig. 4a–d). To ensure that the processing of tonsil into single‐celled suspensions did not affect immunoglobulin release, tonsils were instead cultured as histocultures, either unstimulated or treated with SpeA. Histocultures have a shorter survival time *in vitro*, hence responses were measured after 48 h incubation with SpeA, before onset of tissue necrosis. Tonsil histocultures at 48 h produced concentrations of IgG that were two orders of magnitude greater than tonsil cell suspensions. Similar to results using cell suspensions, a reduction in total IgG was again demonstrated in SpeA‐stimulated histocultures (Fig. 4e).

**Figure 5:**
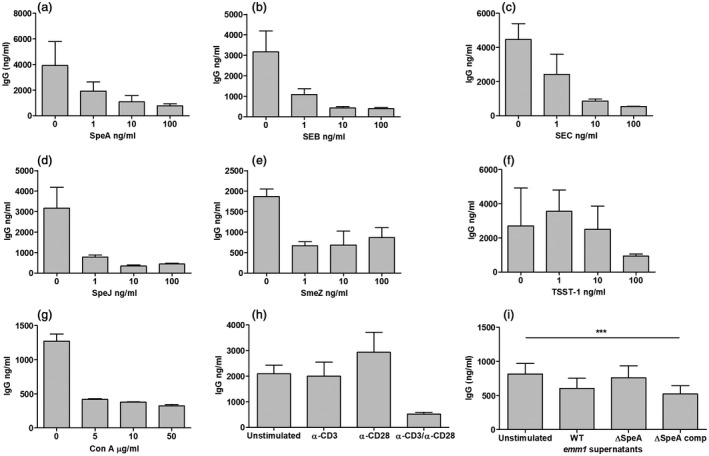
Immunoglobulin (Ig)G production by tonsil cell suspensions is reduced by superantigens and T cell mitogens. IgG release in the presence of different concentrations of bacterial superantigens was tested at 1 week: streptococcal pyrogenic exotoxin (SpeA) (a), staphylococcal enterotoxin B (SEB) (b), staphylococcal enterotoxin C (SEC) (c), streptococcal pyrogenic exotoxin J (SpeJ) (d), streptococcal mitogenic exotoxin Z (SmeZ) and toxic shock syndrome toxin 1 (TSST‐1) (f). For comparison, the T cell mitogen concanavalin (Con) A was tested (g) and T cell stimulation using anti(α‐)CD3 and CD28 antibodies (h). Mean and standard deviation (s.d.) of experimental triplicates from one representative tonsil donor (data representative of experiments using three donors for the different superantigens, two for concanavalin A and α‐CD3/28. IgG release was measured from cells cultured in the presence of *emm1* *S. pyogenes* culture supernatants differing only in SpeA production (i). Supernatants containing SpeA [wild‐type (WT) and SpeA‐complemented, ΔspeAcomp] reduced IgG production, in contrast to supernatant from a SpeA gene deletion mutant (ΔspeA). Mean and standard deviation (s.d.) of five donor tonsils shown, *P* < 0·0001*** (two‐way analysis of variance (anova)].

To determine if this effect was specific to SpeA, or might result from any mitogen‐driven tonsil T cell proliferation, tonsil cell suspensions were stimulated with a range of known superantigens and mitogens (Fig. 5). A reduction in total immunoglobulin was again demonstrated following co‐incubation with other streptococcal superantigens (SpeJ and SmeZ) and staphylococcal superantigens [staphylococcal enterotoxins (SE) B, SEC and toxic shock syndrome toxin‐1 (TSST‐1)] in a dose‐dependent manner (Fig. 5a–f). The effect was also demonstrated with the lectin mitogen concanavalin A (Fig. 5g). Stimulation with CD28 antibody produced an increase in immunoglobulin production by tonsil cell suspensions, while no change was induced by anti‐CD3. However, combined anti‐CD3 and anti‐CD28 resulted in a decrease in IgG production by tonsil cells (Fig. 5h). Bacteria produce a complex mix of factors that together might be expected to increase IgG production by tonsil B cells, notwithstanding any effect of superantigens. However, exposure of tonsil cells to 1% *S. pyogenes* culture supernatant led to a reduction in tonsil IgG which, using supernatants from isogenic strains differing only in SpeA production, could be specifically ascribed to production of SpeA (Fig. 5i). Transferred cytokine‐rich supernatants from SpeA‐stimulated tonsil cell suspensions did not elicit inhibition of total immunoglobulin release when added to unstimulated tonsil cells, and the effect was not reversed by antibodies neutralizing individual cytokines (Supporting information, Fig. 2). The data showed that superantigen‐induced T cell proliferation and activation in the tonsil results in B cell apoptosis and loss of function, manifest as immunoglobulin suppression; this effect was observed in tonsil cell suspensions and histocultures and did not appear to be the direct result of any transferrable soluble mediator.

### Phenotypical change in Tfh cells following SpeA stimulation

**Figure 6:**
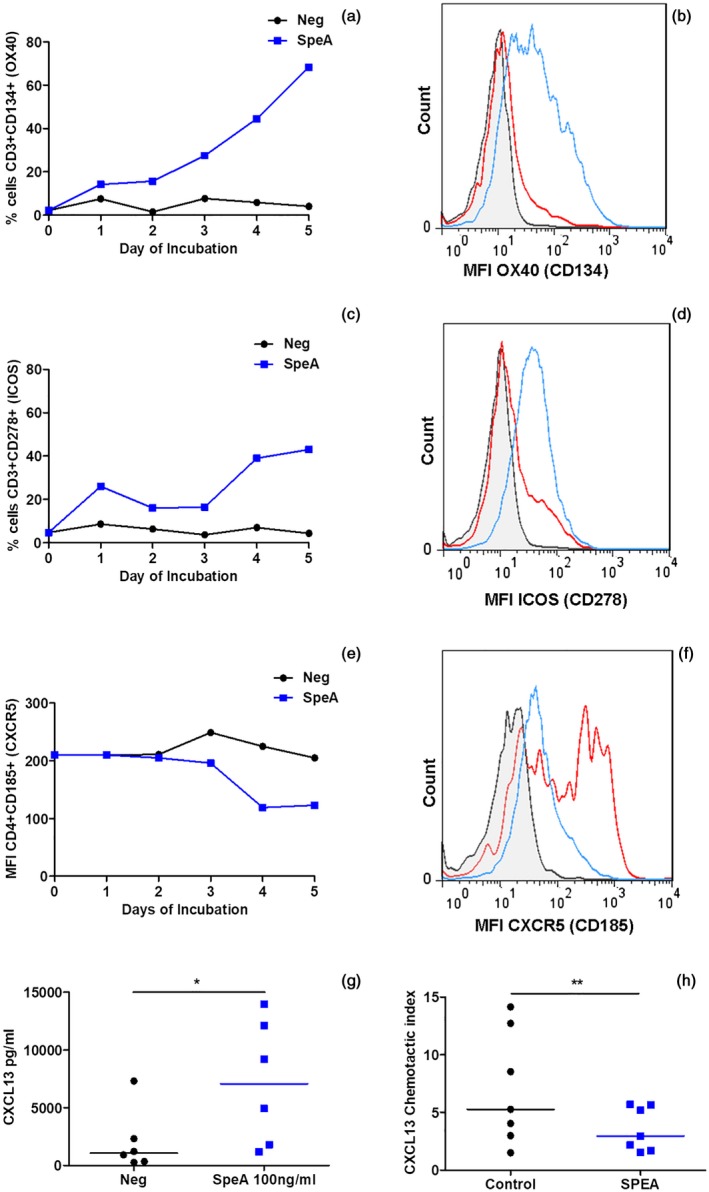
Altered T follicular helper (Tfh) phenotype in streptococcal pyrogenic exotoxin (SpeA)‐stimulated tonsil cells. Tonsil cells stimulated with SpeA (blue squares) showed a marked early increase in the percentage and mean fluorescence intensity (MFI) of CD3^+^ T cells expressing OX40 (CD134, a,b, *P* = 0·029) and inducible T cell co‐stimulator (ICOS) (CD278, c,d (*P* = 0·029) compared with unstimulated controls (black circles) (Wilcoxon’s paired signed‐rank *t*‐test). Four tonsil donors for OX40 and ICOS, representative time–course (days 1–5) and fluorescence activated cell sorter (FACS) plot of gated T lymphocytes are shown from one donor each (day 5); grey‐shaded, isotype control; red line, unstimulated cells; blue line, SpeA 100 ng/ml. In contrast, expression of C‐X‐C motif chemokine receptor (CXCR5) in CD4^+^ cells reduced in response to SpeA compared with unstimulated controls (e,f, *P* = 0·015*, Wilcoxon’s paired signed‐rank *t*‐test). Seven donors for CXCR5, time–course and FACS plot from one representative donor (day 5) as described above. SpeA stimulated tonsil cells showed an increase in chemokine (C‐X‐C motif) ligand (CXCL)13 production (g, *P* = 0·0313 Wilcoxon’s paired signed‐rank *t*‐test) but chemotaxis of tonsil CD4^+^CXCR5^+^ cells toward CXCL13 gradient was reduced by prior co‐incubation with SpeA [h, *P* = 0·005**, two way analysis of variance (anova)].

Tfh cells represent a major proportion of T cells in tonsil suspensions and are necessary for B cell maturation and function in germinal centres. SpeA induced a significant expansion and increase in expression of key Tfh activation markers OX40 (CD134) and inducible T cell co‐stimulator (ICOS) (CD278) (Fig. 6a–d). Although the population of CXCR5 (CD185)‐expressing T cells expanded in response to SpeA, expression of CXCR5 (CD185) reduced over time, despite increase in other activation markers (Fig. 6e,f). To determine if the observed reduction in CXCR5 (CD185) expression was likely to be functionally relevant, CXCL13 (B cell‐attracting chemokine 1) production and CXCL13 chemotaxis assays were undertaken: Despite increased tonsil cell production of CXCL13 in response to SpeA, SpeA‐stimulated tonsil T cells demonstrated reduced chemotactic responses towards CXCL13 compared with controls (Fig. 6g,h).

## Discussion

Primary human tonsil cells demonstrate TCR‐Vβ‐specific clonal T cell expansion and proinflammatory cytokine release in response to streptococcal superantigens. Based in a non‐sterile site, the tonsil is repeatedly exposed to bacterial and other antigens; as such, B cells that reside in germinal centres are primed to release significant amounts of immunoglobulin even at rest, supported by a large population of CXCR5‐expressing Tfh cells that are resident in the tonsil 14. Focusing on the phage‐encoded superantigen SpeA, implicated in the success of the globally successful *emm*/M1 *S. pyogenes* lineage 7, 15, we identified a paradoxical reduction in release of multiple classes of immunoglobulin including IgG, IgA and IgM in response to superantigen. This occurred despite the marked increase in proinflammatory cytokine secretion by tonsil cells. Diminution of tonsil immunoglobulin production was reproduced by all superantigens tested, suggesting a common mechanism of immune subversion for both *S. pyogenes* and *Staphylococcus aureus*, and was recapitulated by anti‐CD28/anti‐CD3. In the more physiological context of bacterial supernatant, that contains mixtures of otherwise potent superantigens, it was possible to assign a more specific role to SpeA, suggesting that SpeA may play a pivotal role in diminution of antibody production either alone or in synergy with other superantigens.

The participating constituent cells in tonsils are markedly different to those in peripheral blood, and cell populations are in close contact. Tonsil T cell populations expanded by SpeA demonstrated an altered phenotype, expressing high levels of the TNF receptor superfamily members OX40 (CD134) and ICOS (CD278), but paradoxically low levels of CXCR5 (CD185) compared with unstimulated controls, with reduced chemotactic responses to the chemokine CXCL13. Taken together, the data point to a specific role for streptococcal superantigens in recruiting and diverting tonsil follicular T helper cells from their primary purpose, thereby undermining antibody response during *S. pyogenes* infection, and conceivably other infections that rely on antibody production in the tonsil. Indeed, a recent report suggested that the presence of superantigen‐associated *Staph. aureus* in tonsil may result in skewing of the tonsil TCR‐Vβ repertoire 16. Expansion of T cells in tonsil cultures was accompanied by a paradoxical absolute reduction in B cells that was associated with evidence of B cell apoptosis.

SpeA was first reported to reduce immunoglobulin production almost 50 years ago; a reduction in immunoglobulin‐secreting B cells was accompanied by a paradoxical increase in spleen weight and 100% expansion in total number of cells in rabbits exposed to SpeA 17. Notwithstanding the recognized poor sensitivity of murine cells to superantigens, both SpeA and SpeC were shown in older reports to suppress murine spleen B cell IgM responses in a manner that required altered activity of T cells, raising the possibility of either reduced T helper cell function or an increase in T suppressor activity 18. While superantigen‐induced T cell‐derived secreted products were reported by some to suppress immunoglobulin‐secreting cells 1, 19, 20, others have reported contact‐dependent T cell‐induced Fas (CD95)‐dependent B cell apoptosis following superantigen exposure in human peripheral blood mononuclear cells, although in our study we did not investigate Fas (CD95)–Fas ligand (CD95L) interactions. In the tonsil cultures used in the current report, reduction in immunoglobulin required co‐incubation of B and T cells and could not be recapitulated by secreted products from T cells, albeit that tonsil cells were exposed to only a 1% SpeA‐exposed tonsil cell medium. This points to a requirement for contact between the effector T cells and apoptosing B cells, at least in the experimental setting, but does not exclude a role for broad disruption of TfH‐B cross‐talk by the cytokine cocktail elicited by superantigen exposure.

Within the tonsil and other solid lymphoid organs, distinct populations of Tfh cells, characterized by expression of CXCR5 (CD185), OX40 (CD134) and PD1 (CD279), are now recognized to underpin B cell viability through migration to germinal centres in response to CXCL13, a chemokine also required for B cell migration to form follicles 14. More recently, regulatory T follicular cells have been described to repress B cell activity, and the net balance between the two T cell populations is believed to underlie B cell activity in lymphoid tissue 21. In the static model used in our study, however, forkhead box P3 (FoxP3) was detected in approximately 5% of T cells, and we therefore conclude that, in the *ex‐vivo* setting described in the current work, classically defined regulatory cells are unlikely to have played a major role. This does not exclude the possibility that superantigen‐stimulated regulatory follicular T cells may play a greater role *in vivo* in lymph nodes draining sites of infection, noting that superantigens are reported to activate regulatory T cells in peripheral blood 22. In this study, the phenotype of T cells in SpeA‐expanded tonsil cell cultures was significantly and consistently altered such that expression of CXCR5 (CD185) reduced, potentially impacting on chemotactic function, while other markers of Tfh activation such as ICOS (CD278) were increased. We speculate that within the tonsil, reduced chemotactic function may render the Tfh cells unable to migrate to germinal centres and promote B cell antibody production, providing one potential explanation for our findings in histocultures, although we did not specifically examine the chemotactic function of an isolated TfH population. It is also possible that the augmented production of CXCL13 by tonsil cells in response to SpeA in some way corrupts the normal homeostasis of B or T cell migration. Notwithstanding these findings, it is clear that the altered phenotype of this T cell population in the presence of superantigens requires further evaluation.

Study of human tonsil cellular responses are necessarily limited by availability of surgical tissue, donor heterogeneity, bacterial contamination of samples and tissue viability, probably accounting for interindividual variation 23. The preponderance of female donors in the current study is unexplained, but could represent a bias in selection for surgical referral or tissue bank consent, factors that could not be controlled for. Nonetheless, we did not observe a difference between males and females. Individual clinical history in tonsil donors may influence response to superantigens, particularly as the donors were adults with history of tonsillitis as the leading indication for surgery. Repeated exposure to streptococcal infection might be expected to lead to immunity to toxins such as SpeA. As many adults have neutralizing antibodies to superantigens 1 and as tonsil cells produce abundant immunoglobulin, the detection of any effect of SpeA within tonsil cell culture is therefore all the more remarkable, underlining the significance of the effects observed.

SpeA was selected as a paradigm of streptococcal superantigens as it is strongly associated with the *emm*1 genotype that has caused disease worldwide, and is historically associated with scarlet fever. However, SpeA is less potent in human systems than other streptococcal superantigens such as SpeJ and SmeZ 8, potentially because SpeA‐responsive TCR‐Vβ subsets are smaller, although may alter with stimulus strength 24. We used SpeA at a concentration that is physiologically relevant, representing typical concentrations achieved in broth culture 11, noting that the concentrations of superantigens within the tonsil during infection have not been characterized.

Since the emergence of invasive group A streptococcal infections in the late 1980s and description of a superantigen‐associated toxic shock‐like syndrome 7, there have been a number of reports that describe superantigen‐related effects in invasive human infection 25, 26. Data showing superantigen‐dependent effects in humanized transgenic mice support a role for superantigens in severe disease pathogenesis and inflammation 1, 9; however, invasive infection is rare, and the evolutionary benefit of superantigen production is most likely to be manifest in more common non‐invasive infections such as tonsillo‐pharyngitis. Recent studies in humanized transgenic mice point to a role for SpeA in permitting nasopharyngeal infection through a mechanism that requires TCR‐Vβ‐specific T cell expansion 27, while others have highlighted impairment of memory B cell responses to first streptococcal skin infections in mice 28. The extent to which the current findings in human tonsils explain the reported phenomena in mice is unclear, noting that the composition of tonsil is quite different to mucosal‐ and nasal‐associated lymphoid tissue. Certainly, if superantigens such as SpeA subvert production of antibody and initiation of adaptive immune response to *S. pyogenes*, this provides a rationale for inclusion of SpeA and other superantigen toxoids in any future *S. pyogenes* vaccine, in order to provide neutralizing immunity against SpeA and other superantigens. A similar approach has been proposed for *Staph. aureus*, where development of natural adaptive immunity may be thwarted by exposure to the B cell superantigen staphylococcal protein A 29.

Leucocytes from the peripheral blood produce limited amounts of immunoglobulin in cell culture; the current work underlines the importance of studying relevant lymphoid tissue when evaluating responses to bacterial pathogens. Indeed, the immunoglobulin released by intact tonsil histocultures exceeded levels produced by cell suspensions by several orders of magnitude. The unexpected findings highlight that even disaggregated tonsil cell suspensions may not faithfully reproduce the cellular interactions occurring in intact lymphoid follicles. The human tonsil provides an excellent model system for study of streptococcal disease that can reduce and replace the use of animals in such research, although cannot completely reproduce the dynamic changes in cell populations that may occur in a draining lymph node. The current work has identified a clear need to further understand the impact of superantigens such as SpeA on development of adaptive immunity to *S. pyogenes*, elucidation of which is likely to require a nuanced approach combining transgenic mouse models and human cells.

## Disclosures

None.

## Supporting information


**Fig.** **S1** **.** Superantigen‐induced tonsil cell TCRVβ profile in different tonsil donors. Fold change from baseline profile is shown following superantigen stimulation for 7d with SpeA (a) and SmeZ (b). Each data point represents a single donor: 6‐8 donors (SpeA), 2‐4 donors (SmeZ). In two donors, only 4 TCRVβ subsets were checked (Vβ2, 8, 11 and 14). Tonsil cell TCRVβ profile for Vβ2, 8, 11 and 14 following *emm*/M1 streptococcal supernatant stimulation in a single tonsil donor confirmed SpeA‐specific TCRVβ changes (c) when comparing supernatants from parent (WT); isogenic *speA* negative (WTΔspeA); and complemented (WTΔspeA comp) strains with unstimulated cells (neg) from the same tonsil donor. Tonsil cell TCRVβ profile following *emm*/M89 supernatant stimulation in a single tonsil donor confirmed SmeZ‐specific changes (d) when comparing supernatants from parent (WT); isogenic *smeZ* negative (WTΔsmeZ); and complemented (WTΔsmeZ comp) strains with unstimulated cultures from the same tonsil donor. Results for the TCRVβ subsets 1, 2, 4, 7.1, 8, 9, 13.2, 18 and 23 only are shown, as there was no alteration from baseline with the other TCRVβ subsets tested.
**Fig.** **S2** **.** Effect of soluble factors on tonsil IgG production. (a) To determine whether SpeA exposed tonsil cells produced a secreted factor that could inhibit IgG production, cell‐free supernatants from SPEA‐exposed tonsil cells were transferred to naive tonsil cell cultures. IgG production by naïve tonsil cells (Negative group, horizontal axis) was unaffected by co‐incubation with 1% culture supernatant transferred from tonsil cells that had been previously exposed to either SpeA 100 ng/ml for 7d (black bars, SPEA SN) or medium only (white bars, Negative SN). Fresh tonsil cultures did however respond to SpeA (SPEA 100 ng/ml) when added directly; IgG after 7d was reduced in all settings. Error bars represent mean + SD. of triplicate IgG levels from one tonsil donor. Data are representative of 2 additional naïve tonsil cultures, using transferred supernatants obtained at different time points. (b) Effect of inhibiting cytokines on tonsil IgG production. Tonsil cultures were either unstimulated (Negative group, horizontal axis) or stimulated with SpeA 100 ng/ml (SPEA 100 ng/ml group, horizontal axis) at the start of culture. The following inhibitory antibodies (10 μg/ml) were added at days 0, 2 and 5 of culture: Negative/normal goat serum, grey bars; goat‐anti IL4, white bars; goat anti‐IL10, black bars; goat anti‐TNF; spotted bars; goat anti‐INF, striped bars. Data show mean and SD of 3 experimental replicates. Data representative of *n* = 2 donors for IL4, *n* = 3 donors for IL10, TNFα and INFγ.
