# Supplementary material for: Streptococcal superantigen‐induced expansion of human tonsil T cells leads to altered T follicular helper cell phenotype, B cell death and reduced immunoglobulin release
Source: Clin Exp Immunol. 2019 Mar 13;197(1):83–94. doi: 10.1111/cei.13282 (PMC6591145; doi:10.1111/cei.13282)

**Supplementary Data**

**Streptococcal superantigen-induced expansion of human tonsil T cells leads to altered T follicular helper cell phenotype, B cell death, and reduced immunoglobulin release.**

Frances J. Davies, Carl Olme, Nicola N. Lynskey, Claire E. Turner^1^, Shiranee Sriskandan*. Department of Medicine, Imperial College London, UK

**Supplementary Figure 1**.

**Superantigen-induced tonsil cell TCRVβ profile in different tonsil donors**. Fold change from baseline profile is shown following superantigen stimulation for 7d with SpeA (A) and SmeZ (B). Each data point represents a single donor: 6-8 donors (SpeA), 2-4 donors (SmeZ). In two donors, only 4 TCRVβ subsets were checked (Vβ2, 8, 11 and 14). Tonsil cell TCRVβ profile for Vβ2, 8, 11 and 14 following *emm*/M1 streptococcal supernatant stimulation in a single tonsil donor confirmed SpeA-specific TCRVβ changes (C) when comparing supernatants from parent (WT); isogenic *speA* negative (WTΔspeA); and complemented (WTΔspeA comp) strains with unstimulated cells (neg) from the same tonsil donor. Tonsil cell TCRVβ profile following *emm*/M89 supernatant stimulation in a single tonsil donor confirmed SmeZ-specific changes (D) when comparing supernatants from parent (WT); isogenic *smeZ* negative (WTΔsmeZ); and complemented (WTΔsmeZ comp) strains with unstimulated cultures from the same tonsil donor. Results for the TCRVβ subsets 1, 2, 4, 7.1, 8, 9, 13.2, 18 and 23 only are shown, as there was no alteration from baseline with the other TCRVβ subsets tested.


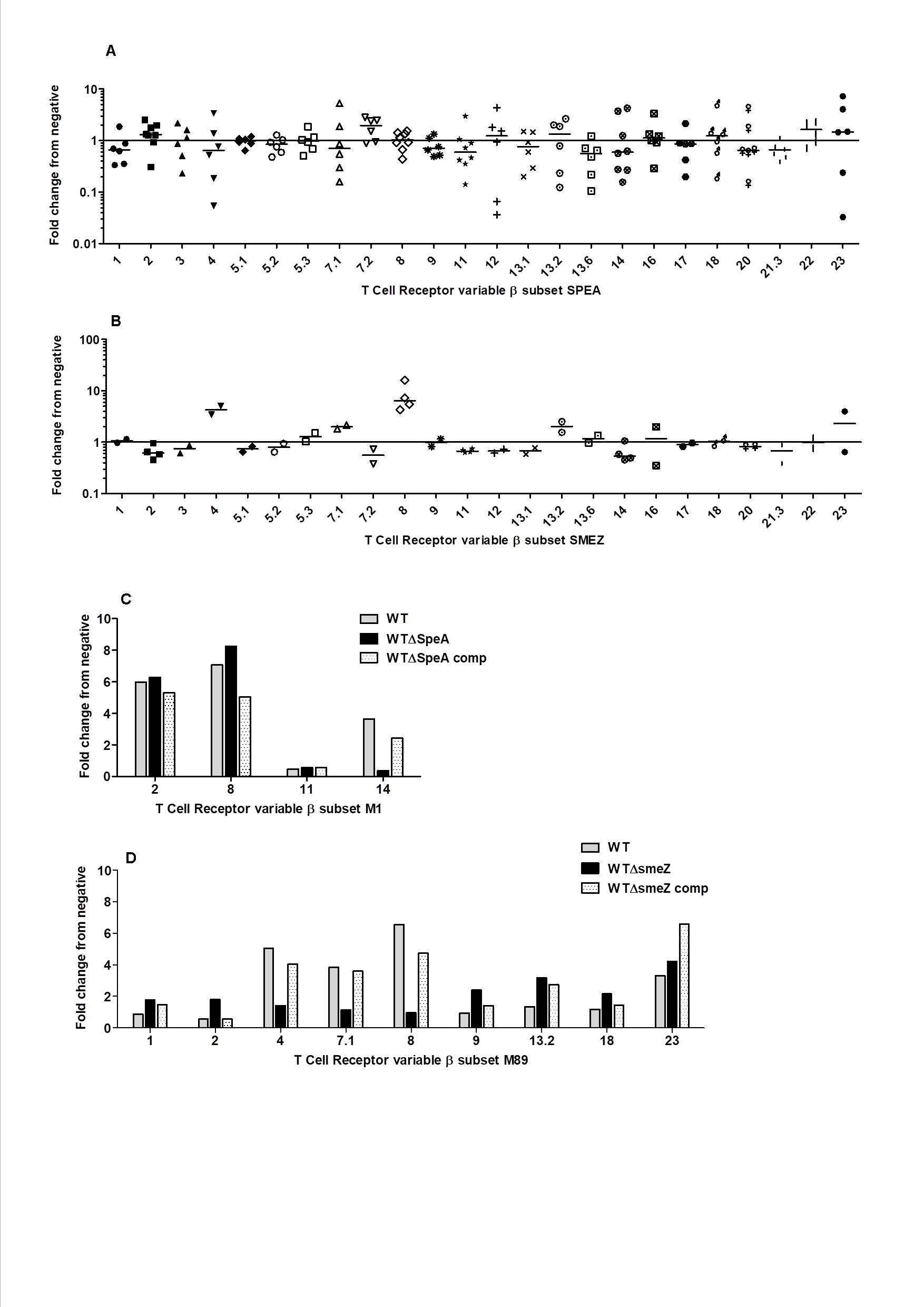


**Supplementary Figure 2**

**Effect of soluble factors on tonsil IgG production.** (A) To determine whether SpeA exposed tonsil cells produced a secreted factor that could inhibit IgG production, cell-free supernatants from SPEA-exposed tonsil cells were transferred to naive tonsil cell cultures. IgG production by naïve tonsil cells (Negative group, horizontal axis) was unaffected by co-incubation with 1% culture supernatant transferred from tonsil cells that had been previously exposed to either SpeA 100ng/ml for 7d (black bars, SPEA SN) or medium only (white bars, Negative SN). Fresh tonsil cultures did however respond to SpeA (SPEA 100ng/ml) when added directly; IgG after 7d was reduced in all settings. Error bars represent mean + SD. of triplicate IgG levels from one tonsil donor. Data are representative of 2 additional naïve tonsil cultures, using transferred supernatants obtained at different time points. (B) Effect of inhibiting cytokines on tonsil IgG production. Tonsil cultures were either unstimulated (Negative group, horizontal axis) or stimulated with SpeA 100ng/ml (SPEA 100ng/ml group, horizontal axis) at the start of culture. The following inhibitory antibodies (10 μg/ml) were added at days 0, 2 and 5 of culture: Negative/normal goat serum, grey bars; goat-anti IL4, white bars; goat anti-IL10, black bars; goat anti-TNF; spotted bars; goat anti-INF, striped bars. Data show mean and SD of 3 experimental replicates. Data representative of N=2 donors for IL4, N=3 donors for IL10, TNFα and INFγ.


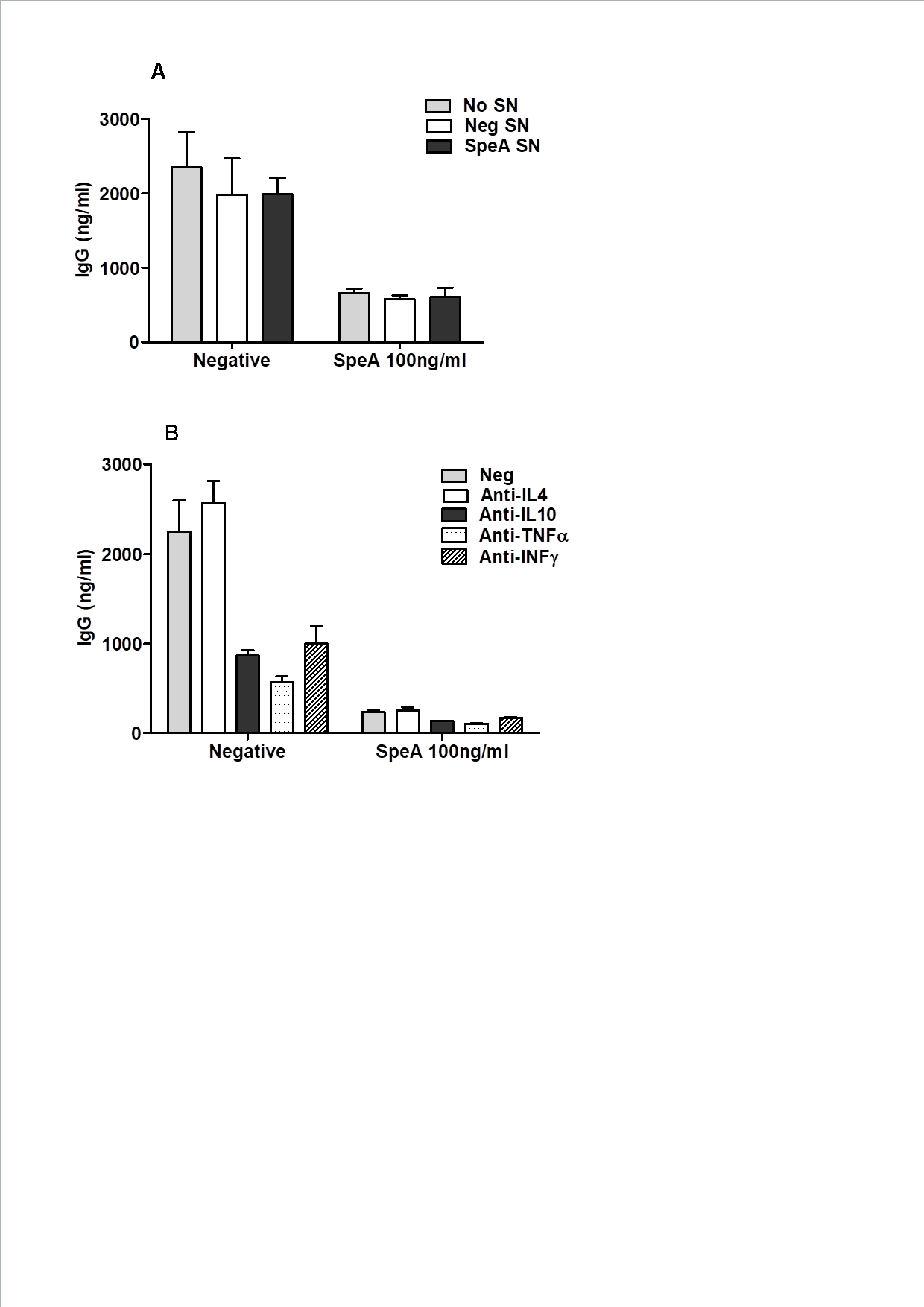

Supplement: Supplementary file 1 — Fig. S1 . Superantigen‐induced tonsil cell TCRVβ profile in different tonsil donors. Fold change from baseline profile is shown following superantigen stimulation for 7d with SpeA (a) and SmeZ (b). Each data point represents a single donor: 6‐8 donors (SpeA), 2‐4 donors (SmeZ). In two donors, only 4 TCRVβ subsets were checked (Vβ2, 8, 11 and 14). Tonsil cell TCRVβ profile for Vβ2, 8, 11 and 14 following emm/M1 streptococcal supernatant stimulation in a single tonsil donor confirmed SpeA‐specific TCRVβ changes (c) when comparing supernatants from parent (WT); isogenic speA negative (WTΔspeA); and complemented (WTΔspeA comp) strains with unstimulated cells (neg) from the same tonsil donor. Tonsil cell TCRVβ profile following emm/M89 supernatant stimulation in a single tonsil donor confirmed SmeZ‐specific changes (d) when comparing supernatants from parent (WT); isogenic smeZ negative (WTΔsmeZ); and complemented (WTΔsmeZ comp) strains with unstimulated cultures from the same tonsil donor. Results for the TCRVβ subsets 1, 2, 4, 7.1, 8, 9, 13.2, 18 and 23 only are shown, as there was no alteration from baseline with the other TCRVβ subsets tested. Fig. S2 . Effect of soluble factors on tonsil IgG production. (a) To determine whether SpeA exposed tonsil cells produced a secreted factor that could inhibit IgG production, cell‐free supernatants from SPEA‐exposed tonsil cells were transferred to naive tonsil cell cultures. IgG production by naïve tonsil cells (Negative group, horizontal axis) was unaffected by co‐incubation with 1% culture supernatant transferred from tonsil cells that had been previously exposed to either SpeA 100 ng/ml for 7d (black bars, SPEA SN) or medium only (white bars, Negative SN). Fresh tonsil cultures did however respond to SpeA (SPEA 100 ng/ml) when added directly; IgG after 7d was reduced in all settings. Error bars represent mean + SD. of triplicate IgG levels from one tonsil donor. Data are representative of 2 additional naïve tonsil cultures, [file CEI-197-83-s001.docx]
